# Titanium Dioxide Nanoparticles (TiO_2_) Quenching Based Aptasensing Platform: Application to Ochratoxin A Detection

**DOI:** 10.3390/toxins7093771

**Published:** 2015-09-22

**Authors:** Atul Sharma, Akhtar Hayat, Rupesh K. Mishra, Gaëlle Catanante, Sunil Bhand, Jean Louis Marty

**Affiliations:** 1 BAE Laboratory, Université de Perpignan Via Domitia, 52 Avenue Paul Alduy, Perpignan 66860, France; E-Mails: p2012407@goa.bits-pilani.ac.in (A.S.); akhtarloona@gmail.com (A.H.); rupeshmishra02@gmail.com (R.K.M.); gaelle.catanante@univ-perp.fr (G.C.); 2 Biosensor Lab, Department of Chemistry, BITS, Pilani- K. K. Birla Goa Campus, Zuarinagar, Goa 403726, India; E-Mail: sgbhand@gmail.com; 3 Interdisciplinary Research Centre in Biomedical Materials (IRCBM), COMSATS Institute of Information Technology (CIIT), Lahore 54000, Pakistan; 4 Department of Biosciences and Biotechnology, Banasthali University, Rajasthan 304022, India

**Keywords:** titanium dioxide nanoparticles, fluorescently labeled aptamer, aptamer assay, ochratoxin A, beer, quenching

## Abstract

We demonstrate for the first time, the development of titanium dioxide nanoparticles (TiO_2_) quenching based aptasensing platform for detection of target molecules. TiO_2_ quench the fluorescence of FAM-labeled aptamer (fluorescein labeled aptamer) upon the non-covalent adsorption of fluorescent labeled aptamer on TiO_2_ surface. When OTA interacts with the aptamer, it induced aptamer G-quadruplex complex formation, weakens the interaction between FAM-labeled aptamer and TiO_2_, resulting in fluorescence recovery. As a proof of concept, an assay was employed for detection of Ochratoxin A (OTA). At optimized experimental condition, the obtained limit of detection (LOD) was 1.5 nM with a good linearity in the range 1.5 nM to 1.0 µM for OTA. The obtained results showed the high selectivity of assay towards OTA without interference to structurally similar analogue Ochratoxin B (OTB). The developed aptamer assay was evaluated for detection of OTA in beer sample and recoveries were recorded in the range from 94.30%–99.20%. Analytical figures of the merits of the developed aptasensing platform confirmed its applicability to real samples analysis. However, this is a generic aptasensing platform and can be extended for detection of other toxins or target analyte.

## 1. Introduction

Nanotechnology, a promising approach for the development of rapid, selective and specific bioanalytical methods for detection of contaminants and drug molecule in the area of agriculture and food industry [1,2]. Development of bifunctionally modified nanoparticle and nanoconjugates of ligand molecule has provided notable transition of biological and biochemistry knowledge into applied science and bioanalytical applications [3,4,5,6,7,8,9,10,11]. Recently, the development of aptamer assay based on electrochemical [12], fluorescence [13], piezoelectric [14], chemiluminescence [15] and calorimetric [16] signal generation has received an extensive consideration due to high binding capacity, selectivity and fluorophore labeling characteristics of aptamer. Amongst all approaches, the fluorescence signaling based aptamer assay has attained prominent attention due to their simplicity, applicability of diverse measurement methods and high-throughput analysis [17]. In the recent research trend, the nanostructures such as graphene or graphene oxide (GOx) [18,19,20], carbon nanotubes (CNT) [21], carbon nanoparticles (CNPs) [22], gold nanoparticles (GNPs) [23,24] and silica nanoparticles (SNPs) [25] have been explored for development of fluorescence resonance energy transfer (FRET) or quenching based aptasensing platform for various applications such as DNA hybridization studies, detection of nucleic acid and toxins. Encouragingly, these nanostructures eliminates the selection hindrance of fluorophore-quencher sequence in nanostructures based fluorescence quenching assay associated with classical quenching (FDNA-QDNA) based aptamer assay [21,26]. The applicability of these nanostructures as a quencher eliminates the major disadvantage of dual labeling of aptamer in comparison to the conventional molecular beacons and averts the expensive analysis. Nanomaterials possessing quenching efficiency have been already investigated. However, the salt induced aggregation of nanoparticle, pH, surface charge and the presence of remnant components from synthesis process may affect the interaction between fluorophore labeled aptamer recognition element and nanostructure [23,27]. Several research groups have addressed the consequences of nonspecific adsorption of analyte on the nanostructure surface (such as GOx or CNTs), which could decrease the sensing performance of assay by preventing the interaction between nanostructure surface and aptamer. Functionalization or surface protection of these nanostructures (CNTs and GOx) improves the sensing performance by decreasing non-specific adsorption of target analyte [18,28]. Meanwhile, the surface modification can also decrease the quenching efficiency in FRET or quenching based aptamer assay due to increased hindrance of interaction between aptamer and nanostructure surface. Recently, the titanium dioxide nanoparticles (TiO_2_), naturally occurring nanomaterial has attained considerable attention in development of new bioanalytical methods due to their biocompatible and structural features [29]. Surface adsorption capacity, drug detecting element and quenching potential of TiO_2_ have already been reported [27,30]. Recently, Li *et al.*, (2013) reported a hybrid TiO_2_ system for simultaneous determination of biomolecule and controlled drug release [31]. However, to date, no reports have been published on TiO_2_ quenching based aptasensing platform for detection of biomolecules.

In the present work, the quenching efficiency of TiO_2_ was explored and employed for the development of quenching based aptasensing platform. Herein, we demonstrate, the development of TiO_2_ quenching based aptamer assay for biomolecules detection using TiO_2_-FAM quenching system. The adopted strategy employed the use of TiO_2_ as quencher and fluorescently labeled (FAM-modified) aptamer both as fluorophore and recognition elements for target molecule detection. Later on, an application of sensing platform was demonstrated for the detection of OTA. OTA was selected as a model target analyte due to its presence in common food stuffs, highly toxic and potential carcinogenic effects. The demonstrated system is a new addition to the state of the art in OTA analysis. The existing literature reveals that we could able to achieve a better sensitivity in terms of limit of detection (LOD) for OTA using developed assay, the comparisons are summarized in Table 1. The developed sensing platform shows promising potential, being capable of detecting OTA below the maximum residue limit (MRL) of OTA, *i.e.*, 2 µg/kg.

**Table 1:** Comparison with earlier reported aptamer assay for OTA detection.

S. No.	Materials	Methods	Linear range	LOD	Reference
1.	Single walled carbon nanotubes (SWNTs)	Fluorescence	25–200 nM	24.1 nM	[32]
2.	Graphene oxide: Bare grapheme PVP coated graphene oxide	Fluorescence	2–35 µM 50–500 nM	1.9 µM 21.8 nM	[18]
3.	Nanographite: Bare nanographite DNase catalyzed amplification	Fluorescence	2–50 µM 0.02–0.4 µM	2 µM 20 nM	[33]
4.	Terbium (Tb^3+^)	Fluorescence	0.1–1 ng/mL	20 pg/mL	[34]
5.	Molecular beacons	Fluorescence	1–100 ng/mL	0.8 ng/mL	[35]
6.	Gold nanoparticles	Colorimetric	20–625 nM	20 nM	[36]
7.	Titanium dioxide nanoparticles (TiO_2_)	Fluorescence	1.5 nM–1 µM	1.5 nM	Present work

## 2. Results and Discussion

### 2.1. Design and Strategy Adopted for Aptasensing Platform

**Figure 1:**
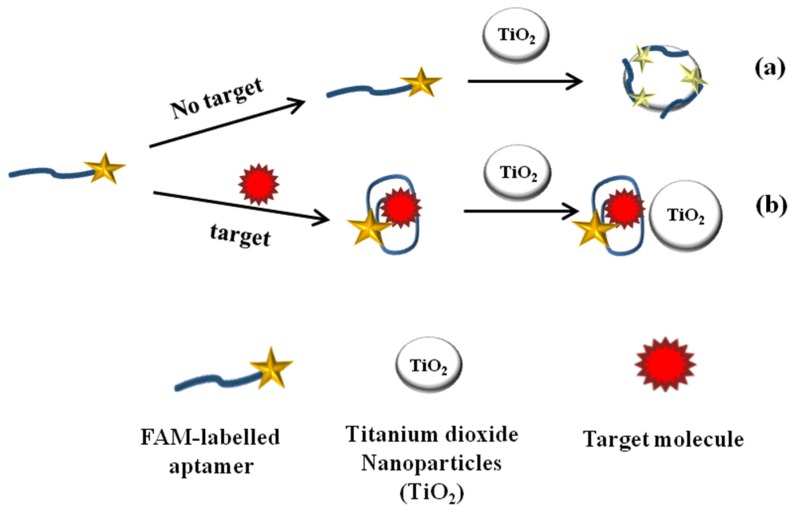
Schematic representation of TiO_2_ quenching based sensing platform for target molecule detection. (**a**) In the absence of target analyte, adsorption of FAM-labeled aptamer on TiO_2_ surface led fluorescence quenching; (**b**) In the presence of target analyte, the anti-parallel G-quadruplex structure form decrease adsorption and fluorescence recovered.

In the present work, we demonstrate the development of TiO_2_ quenching based aptasensing platform for detection of target molecule based on TiO_2_-FAM quenching mechanism as depicted in Figure 1. As shown in the Figure 1a, in the absence of a target molecule, adsorption of FAM-labeled aptamer on TiO_2_ surface results in fluorescence quenching of labeled aptamer. The quenching mechanism of TiO_2_-FAM system can be attributed to the large band gap semiconductor behavior of TiO_2_ and the electrostatic interaction involving Ti-O bond between TiO_2_ and FAM, resulting fluorescence quenching through the acceptance of electrons from excited FAM molecules [30,37,38]. In the presence of a target molecule, the target induces conformational change in aptamer’s structure led to the formation of anti-parallel G-quadruplex structure, which decreases the adsorption and weakens the quenching interaction between FAM and TiO_2_. These induced conformational changes in aptamer structure results in significant recovery of fluorescence in comparison to the fluorescence quenched in the absence of target molecules as shown in Figure 1b. Thus, by monitoring the degree of fluorescence recovered, the presence of target molecule could be measured quantitatively.

### 2.2. UV Characterization of Aptamer-TiO_2_ Complex

**Figure 2:**
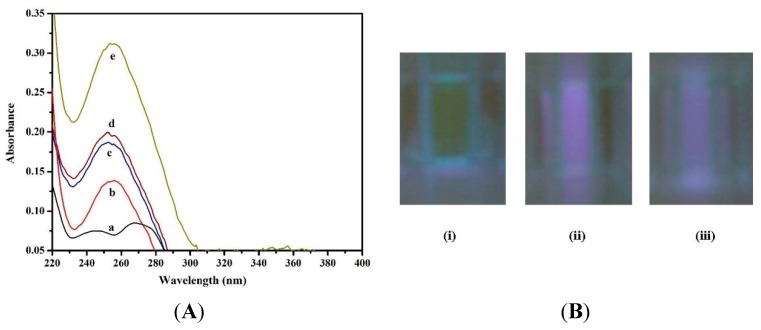
(**A**) UV absorption spectra of (a) TiO_2_; (b) Aptamer; (c) Aptamer-target complex (in absence of TiO_2_); (d) Aptamer-target complex (in presence of TiO_2_); and (e) Aptamer-TiO_2_ complex; (**B**) Fluorescence imaging of (i) FAM-aptamer; (ii) aptamer-TiO_2_; and (iii) aptamer-target complex (in presence of TiO_2_).

UV spectral measurements were performed to characterize the interaction between aptamer and TiO_2_. Figure 2a, the absorption spectrum showed the characteristic absorption maxima of aptamer at 255 nm as shown in (curve b). Figure 2a strongly suggests that the TiO_2_ does not exhibit characteristics UV absorption at 255 nm (curve a). On addition of TiO_2_ (150 µg/mL), the UV absorbance of aptamer significantly enhance without change in peak position (curve e) at 255 nm. Thus, the obtained results emphasize the typical characteristic features of aptamer-TiO_2_ adsorption complex and electrostatic interaction [39]. In the presence of target molecule, the UV absorption decreases significantly (curve d), indicating target induced conformational changes in aptamer structure, which resist the aptamer to be adsorbed on TiO_2_ surface. The UV absorption of aptamer-target complex in the presence and absence of TiO_2_ showed similar type of pattern as shown in (curve c and d). Thus, the obtained results confirm that the fluorescence recovery was due to the formation of target-aptamer quadruplex, resulting increase in distance between FAM and TiO_2_. Additionally, the UV-Vis absorption results are summarized in Table S1.

### 2.3. Characterization of Aptamer-TiO_2_ Complex by Fluorescence Imaging

Figure 2b, the FAM-labeled aptamer exhibits strong fluorescence due to the presence of FAM molecules as shown in Figure 2b(i). Upon addition of TiO_2_, the fluorescence quenching is characterized by change in fluorescence intensity (green to blue), as shown in Figure 2b(ii). The change in fluorescence confirms the quenching interactions between aptamer and TiO_2_. In the presence of a target, the decrease in fluorescence intensity (blue intensity) was obtained, which is a strong evidence of target triggered formation of aptamer-target complex. The target induced conformational change resists the adsorption of aptamer on TiO_2_ surface and fluorescence recovery, labeled as (iii) in Figure 2b.

### 2.4. Optimization of Experimental Variables

**Figure 3:**
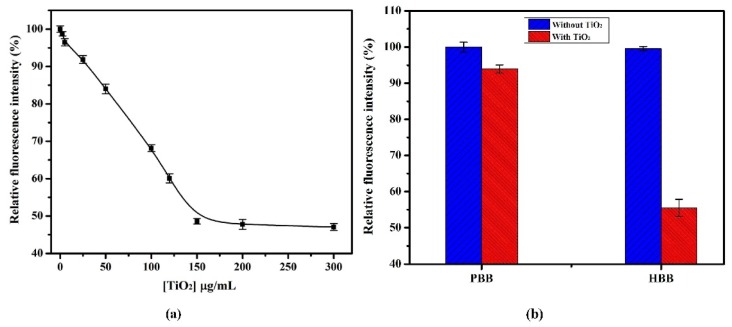
(**a**) Quenching effect of TiO_2_ on fluorescence intensity of FAM-labeled aptamer (final concentration 2.0 µM) in HBB at pH 7.4; (**b**) Effect of HBB and PBB at pH 7.4 on quenching performance of TiO_2_ (final concentration, 150 µg/mL). The error bars were obtained from three parallel experiments.

Experimental variables, which could artifacts the sensing strategy was carefully investigated. As shown in Figure S1, the FAM-labeled aptamer at 2.0-μM concentration exhibits enough fluorescence (λex 485 nm; λem 538 nm) to further investigate the quenching potential of TiO_2_. Figure 3a shows that the fluorescence intensity of FAM-labeled aptamer gradually decreased with increasing concentration of TiO_2_ ranging from 2.5 to 150 μg/mL in HEPES binding buffer (HBB) at pH 7.4. No increase in quenching efficiency was observed with further increase in TiO_2_ concentration thus, 150 μg/mL TiO_2_ was used as an optimum concentration in further experiments. As shown in Figure 3b, the maximum quenching was observed in HBB at pH 7.4, in comparison to the phosphate binding buffer (PBB). Non-specific adsorption of phosphate ions on the surface of TiO_2_ in PBB hinders the adsorption of aptamer, which results in decreased quenching [27,40].

**Figure 4:**
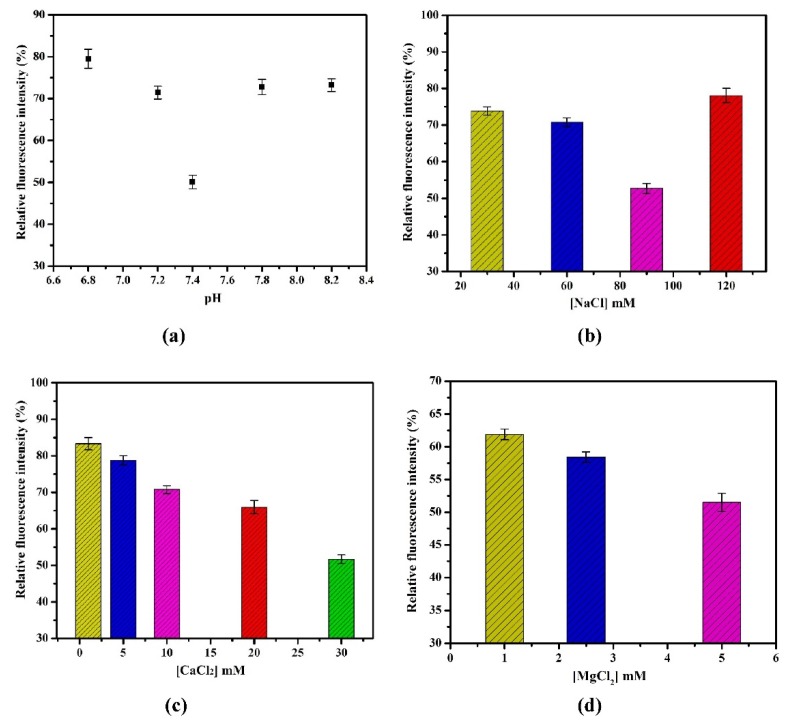
(**a**) Effect of pH (6.8–8.2) in HBB on quenching performance of TiO_2_; (**b**) Effect of NaCl concentrations (30, 60, 90 and 120 mM) on quenching performance of TiO_2_; (**c**) Effect of CaCl_2_ concentrations (1, 5, 10, 20 and 30 mM) on quenching performance of TiO_2_; (**d**) Effect of MgCl_2_ concentrations (1, 2.5 and 5 mM) on quenching performance of TiO_2_. The error bars were obtained from three parallel measurements.

In the Figure 4a, maximum quenching was observed at pH 7.4 in HBB. The change in pH leads to the variation in surface charge of nanoparticles, which may cause the aggregation or render the electrostatic interaction of TiO_2_, consequently decrease in fluorescence quenching [27,41]. Optimization of ionic concentration is highly important, which significantly affects the binding affinity of aptamer with target and quenching efficiency of TiO_2_. To determine the maximum binding affinity of aptamer and evaluation of quenching efficiency, different salt concentrations were tested. As demonstrated in Figure 4b, the fluorescence intensity decreased with increase in concentration of Na^+^ salt (30–90 mM). Maximum quenching was obtained at 90 mM. Figure 4b shows, at much higher concentration of Na^+^ salt (120 mM), fluorescence intensity further increased ,which is strong indication that non-specific adsorption of Na^+^ on TiO_2_ decreases the fluorescence quenching. In addition, divalent metals which increase the binding affinity of aptamer to OTA and acting as ionic bridge enhances the electrostatic interaction in between aptamer and TiO_2_ was also evaluated [18]. As shown in Figure 4c,d, the fluorescence intensity is decreased with increase in concentration of Ca^2+^ and Mg^2+^ salts from 1–30 and 1–5 mM, respectively. To avoid excess salt concentration, higher concentration has not been tested because these concentrations are optimal for binding interaction [42].

Thus, based on the obtained results, it is possible to construct TiO_2_ quenching based fluorescence aptamer assay. Under optimized experimental conditions, the applicability of sensing platform was demonstrated for the detection of OTA as model analyte. In the presence of OTA, the target induced interaction and formation of G-quadruplex structure formation resists the interaction of FAM and TiO_2_, results in fluorescence recovery which quenched in the absence of OTA as shown in Figure S2.

### 2.5. Fluorescence Aptamer Assay for OTA Detection

**Figure 5:**
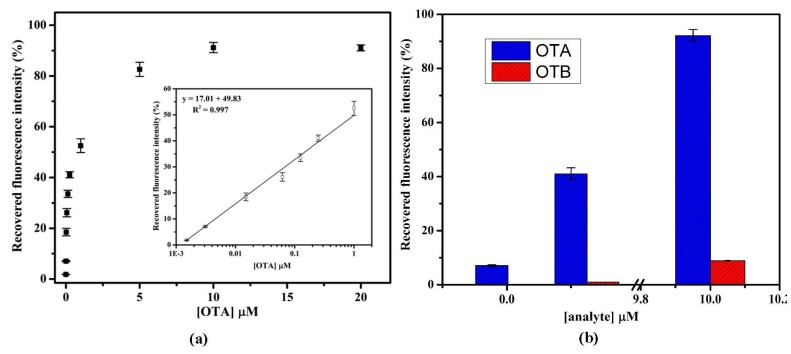
(**a**) Calibration graph for OTA analysis in HBB, pH 7.4 showing recovered fluorescence intensity percentage against OTA concentration. Inset showing the linear fit graph; (**b**) Bar graph representation of specificity studies of aptamer with OTB. Error bars were obtained from three parallel experiments.

Under optimized experimental conditions, an aptamer assay was performed for the detection of OTA. Figure 5a presents the calibration curve of recovered fluorescence intensity percentage with a dynamic detection range of 1.5 nM to 20 µM. As shown in Figure 5a, the % recovery increased with increase in OTA concentration. The calibration curve was fitted using a linear equation and a line of equation obtained was *y* = 17.01*x* + 49.83 with *R*^2^ = 0.997. The good linearity was also obtained ranging from 1.5 nM to 1.0 μM with LOD of 1.5 nM with % R.S.D. = 3.65 (*n* = 3). The LOD was calculated based on the standard deviation of baseline signal. Similarly, the limit of quantification (LOQ) was calculated and found to be 3.1 nM. The obtained results suggest that the fluorescence recovery was due to the interaction of aptamer with OTA.

### 2.6. Selectivity and Specificity of Aptamer Assay

To investigate the selectivity of proposed assay platform that the fluorescence response was due to the specific interaction between aptamer and OTA. The specificity test was performed in the presence of 0.0031, 0.25 and 10.0 µM of OTB spiked in binding buffer. As shown in the Figure 5b, the relative recovered fluorescence response histogram of OTB (red bar) was found to be more accurate with less than 10% (*n* = 3) response of OTA (blue bar). These results confirmed that the fluorescence recovery obtained with OTA was virtually due to specific aptamer and OTA complex formation. The obtained results are summarized in Table S2. This high selectivity of assay could be attributed to the specific aptamer, which results in highly selective binding to OTA.

### 2.7. Analytical Performance of Aptamer Assay for OTA

The repeatability and reproducibility of aptamer assay were investigated by intra and inter day precision experiments. The intra and inter day precision were evaluated at 0.25 µM OTA concentration at optimized experimental conditions. Similarly, control measurements were performed with each assay to calculate the % recovery. Intraday precision with relative standard deviation of 2.89% (*n* = 3) was calculated, indicating a very good reproducibility of aptamer assay. A % RSD from 1.74 to 6.50 (*n* = 3) was calculated from interday analysis, which confirms that the results are reproducible. The obtained results are summarized in the Table 2.

**Table 2:** Analytical and recovery performance of developed assay for detection of OTA.

Interday Analysis Performance of Fluorescence Aptamer Assay					
Days	OTA (μM)		Recovered FL Intensity (a.u.)	Mean ± S.D.	% R.S.D.
Day-1	0.25		17.78	17.78 ± 0.31	1.74
Day-2	0.25		16.76	16.76 ± 1.09	6.50
Day-3	0.25		17.67	17.67 ± 0.62	3.51
**Intraday Analysis Performance of Fluorescence Aptamer Assay**					
**OTA [μM]**	**Recovered FL Intensity (a.u.)**			**Mean ± S.D.**	**% R.S.D.**
	**Response-1**	**Response-2**	**Response-3**		
0.25	17.78	17.02	18	17.6 ± 0.51	2.89
**Recovery Calculation of OTA Analysis from Spiked Beer Sample**					
**Beer Sample**	**OTA Added (µM)**	**OTA Found (µM)**	**Mean ± S.D.**	**% RSD**	**% Recovery**
**1.**	0.0031	0.0029	0.0029 ± 0.0001	3.44	94.30
**2.**	0.2500	0.2480	0.2480 ± 0.0030	1.21	99.20
**3.**	1.0000	0.9910	0.9910 ± 0.0100	1.00	99.10

### 2.8. Application of Aptamer Assay to Beer Sample

To further investigate the feasibility of the developed sensing platform, the detection of OTA was performed in beer sample. The recoveries were performed at three different concentrations, *i.e.*, 0.0031, 0.25 and 1.0 µM of OTA spiked in beer sample. Controls were prepared by adding the same amount of OTA in binding buffer. The recoveries were found in good agreements, ranging from 94.30%–99.20% (*n* = 3). The obtained results are summarized in Table 2, as percentage recovery (% recovery). The obtained results prove the reliability and suitability of developed assay for real matrix.

## 3. Experimental Section

### 3.1. Materials and Reagents

Titanium dioxide nanoparticles (TiO_2_) with the dimension of 25 nm were synthesized and provided by PROMES Laboratory, UPVD-Perpignan, France. HEPES sodium salt was purchased from M/s Fisher Scientific (Fair Lawn, NJ, USA). Sodium phosphate dibasic (Na_2_HPO_4_), potassium phosphate monobasic (KH_2_PO_4_), calcium chloride (CaCl_2_), potassium chloride (KCl), magnesium chloride (MgCl_2_) and sodium chloride (NaCl) were procured from M/s Sigma-Aldrich (St. Quentin Fallavier Cedex, France). OTA, derived from *Aspergillus ochraceus*, was purchased from M/s Sigma Aldrich (St. Quentin Fallavier Cedex, France). OTB, derived from *Aspergillus ochraceus*, was procured from M/s Santa Cruz Biotechnology (Heidelberg, Germany). The beer sample was purchased from local market in Perpignan, France. Deionized Milli-Q water (Millipore, Bedford, MA, USA) was used for preparation of reagents throughout the experiments. The aptamer sequences highly specific to OTA, which form the anti-parallel G-quadruplex structure upon binding to OTA was selected from the reported literature by Cruz-Aguado and Pennar [42]. The dissociation constant (K_d_) value of 0.2 µM between aptamer and the target molecule (OTA) has been calculated and already reported. The fluorophore labeled anti-OTA aptamer (3′FAM-modified) specific to OTA was synthesized and purified by M/s Microsynth (Schützenstrasse, Balgach, Switzerland). The specific sequence of FAM-labeled anti-OTA aptamer used was: 5′-GAT CGG GTG TGG GTG GCG TAA AGG GAG CAT CGG ACA-FAM-3′.

### 3.2. Instrumentation

Fluorescence measurements were carried out using Fluoroskan Ascent FL 2.6 (M/s Thermo Life Sciences, Cergy Pontoise, France) equipped with Ascent software version 2.6 for fluorimetric measurement. UV-visible spectrophotometer (UV-1800, Shimadzu, Japan) equipped with the TCC controller (TCC 240 A) was utilized to measure the absorption characteristics of FAM-labeled aptamer and TiO_2_ complex. Standard (96 microwells) black plates (Thermo Fisher Scientific, Roskilde, Denmark) were used for fluorescence measurement.

### 3.3. Solution Preparation

HEPES binding buffer (HBB, 25 mM) was prepared by dissolving appropriate amount of HEPES containing 30 mM CaCl_2_, 90 mM NaCl, 1.35 mM KCl and 5 mM MgCl_2_ in deionized Milli-Q water. The pH of the buffer was adjusted to 6.8–8.2. Similarly, PBB (10 mM) was prepared by dissolving an appropriate amount of Na_2_HPO_4_, KH_2_PO_4_, containing 5 mM MgCl_2_, 1.35 mM KCl and 60 mM NaCl. The stock solution of FAM-labeled anti-OTA aptamer was prepared in the sterilized deionized distilled water. Pretreatment of aptamer was done (preheated at 85 °C for 5 min and 4 °C for 5 min) before use. For quenching measurements, known amounts of TiO_2_ were dissolved in HBB and further diluted to investigate the quenching efficiency. For preparation of standards, a stock solution of OTA (1 mg/mL) was prepared in methanol and further diluted in HBB for preparation of calibration curve. Similarly, for specificity studies of aptamer, OTB standard solutions were prepared and diluted in HBB. All the working solutions were prepared freshly before use and stored at 4 °C when not in use.

### 3.4. Quenching Measurement by TiO_2_

Prior to quenching measurements, aptamer was pretreated by heating at 85 °C for 5 min and 4 °C for 5 min in PCR thermocycler. A 30 µL volume of FAM-labeled aptamer (final concentration, 2.0 µM) and 10 µL of TiO_2_ (final concentrations, 2.5 to 300 µg/mL) were mixed with 110 µL buffer. The mixture was kept for 1 min at room temperature. The quenching measurements (λex 485 nm; λem 538 nm) were performed continuously for 1 h at each 5 min interval. To calculate the quenching percentage, the control measurements were performed similarly without addition of TiO_2_ and keeping the reaction volume constant (150 µL).

### 3.5. UV Characterization of Aptamer-TiO_2_ Complex

The characterization of aptamer, aptamer-target and aptamer-TiO_2_ complex formation was performed by UV measurements in the range of 200–400 nm. UV absorption of 1 mL solution of FAM labeled aptamer (0.05 µM) was measured in the absence and presence of TiO_2_. Two microliters of (150 µg/mL) TiO_2_ and OTA (0.25 µM) were added individually and mixed properly with aptamer before further measurements.

### 3.6. Fluorescence Aptamer Assay of OTA

For the quantitative measurement of target analyte OTA, FAM-labeled anti-OTA aptamer (final concentration, 2.0 µM) was allowed to mix with the different concentrations of OTA and incubated for 1 h (unless it optimized for 1 h). Then, 10 µL of TiO_2_ (final concentration, 150 µg/mL) was added, mixed properly and incubated for 1 min at room temperature. Fluorescent measurements were carried out using Fluoroskan Ascent FL 2.6. Control measurements were achieved without the addition of OTA in binding buffer under same experimental conditions. Quenching efficiency was calculated using the following equation:

Q (%) = [1 − F_o_/F] (1)

where, F_o_ and F were the fluorescence intensity in the absence and presence of target analyte, respectively [43].

### 3.7. Beer Sample Preparation

The commercial beer sample was purchased from the local market of Perpignan, France. In fluorimetric measurement, the fluorescent compound present in the beer may lead to the false signal and may decrease the assay performance. To avoid such problems, a simple strategy was adopted to obtain the best recovery analysis in spiked beer samples. In brief, the known high concentration of OTA was spiked in beer sample. Afterwards, the spiked samples were degassed for 30 min to avoid rapid foaming. The pH of spiked sample was adjusted to 7.4 and filtered through 0.45 µm Minisart non-pyrogenic filters (Sartorius Stedim Biotech, Goettingen, Germany). Further, the subsequent dilutions were made with unspiked beer sample (4 times) to obtain the different concentration of OTA (0.0031, 0.25 and 1.0 µM) in assay volume (150 µL). Dilution factor was taken into consideration for recovery calculation.

## 4. Conclusions

We have developed, the TiO_2_ quenching based aptasensing platform for toxin/target molecule detection (OTA as model analyte) using TiO_2_-FAM quenching system. The target induced conformational changes render the adsorption of FAM-labeled aptamer on TiO_2_ surface, results in fluorescence recovery with LOD and LOQ of 1.5 and 3.1 nM, respectively. The major advantage of proposed platform is the use of cheaper nanomaterial (TiO_2_) and the developed aptasensing platform is not limited to the OTA analysis. In a word, the developed aptasensing platform can be extended for detection of other analyte or toxins with the availability of target specific fluorescently labeled aptamer. The analytical figures of merits proved the acceptability of sensing platform for other food samples or matrices.

## Supplementary Materials

Supplementary materials can be accessed at: http://www.mdpi.com/2072-6651/7/9/3771/s1.
