# Supplementary material for: Titanium Dioxide Nanoparticles (TiO2) Quenching Based Aptasensing Platform: Application to Ochratoxin A Detection
Source: Toxins (Basel). 2015 Sep 22;7(9):3771–84. doi: 10.3390/toxins7093771 (PMC4591649; doi:10.3390/toxins7093771)
Supplement: Supplementary file 1 [file toxins-07-03771-s001.pdf]

Supplementary Information

Table S1. UV-Vis absorption characterization of TiO_2-aptamer complex at λ_{max} of 255 nm in HBB (pH 7.4).

S. No	Description	Absorbance
1.	Aptamer	0.139
2.	Aptamer-OTA	0.186
3.	Aptamer-OTA-TiO_2	0.195
4.	Aptamer-TiO_2	0.312

Selectivity of proposed TiO_2 based platform for OTA detection. The selectivity of proposed assay for OTA was also evaluated using three different concentration of Ochratoxin B (OTB). As can be clearly seen in Table S2, the obtained fluorescence response of OTB were accurate and less than 10% in comparison to OTA. Obtained results were attributed to the high selectivity of aptamer used for analysis of OTA.

Table S2. Specificity performance of TNPs assay for OTA detection.

Concentration	OTA Recovered FL Intensity (%)		OTB Recovered FL Intensity (%)		% Response of OTB
(μM)	Mean ± S.D. (n = 3)	% R.S.D.	Mean ± S.D. (n = 3)	% R.S.D.	($F_{OTB}/F_{OTA} \times 100$)
0.0031	7.11 ± 0.269	3.78	0.014 ± 0.0005	3.57	0.197
0.25	41.07 ± 2.199	5.35	0.961 ± 0.0515	5.36	2.339
10	92.22 ± 2.137	2.32	8.871 ± 0.2055	2.32	9.619

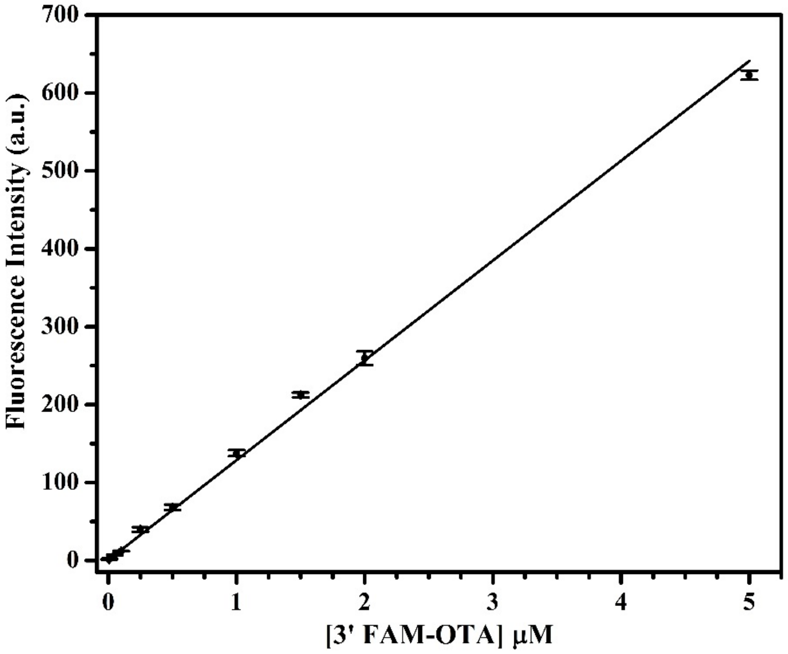

Figure S1. Optimization of FAM-labeled anti-OTA aptamer. The error bars were obtained from their parallel measurements.

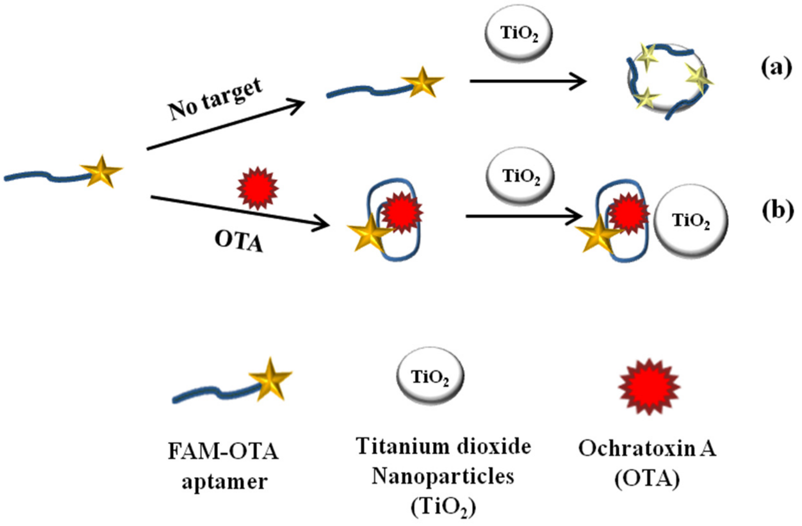

Figure S2. Schematic representation of TiO_2 quenching based aptamer assay for ochratoxin A (OTA). (**a**) Adsorption of FAM-labeled anti-OTA aptamer on TiO_2 surface causes fluorescence quenching; (**b**) In presence of OTA, the formation of anti-parallel G-quadruplex structure formation resist further adsorption and the fluorescence is recovered.
